# Predictors for vancomycin resistant *Enterococcus faecium* transforming from colonization to infection: a case control study

**DOI:** 10.1186/s13756-019-0647-7

**Published:** 2019-12-02

**Authors:** Pao-Yu Chen, Yu-Chung Chuang, Jann-Tay Wang, Wang-Huei Sheng, Yee-Chun Chen, Shan-Chwen Chang

**Affiliations:** 1 Department of Internal Medicine, National Taiwan University Hospital, No. 7 Chung-Shan South Road, Taipei, Taiwan 100; 2 Graduate Institute of Clinical Medicine, College of Medicine, National Taiwan University, Taipei, Taiwan; 3 National Institutes of Infectious Diseases and Vaccinology, National Health Research Institutes, Miaoli, Taiwan; 4 College of Medicine, National Taiwan University, Taipei, Taiwan

**Keywords:** Vancomycin-resistant *Enterococci*, Active surveillance, Pulsed-field gel electrophoresis, Multilocus sequence typing, Clonal complex 17

## Abstract

**Background:**

Little is known about risk factors for subsequent infections among vancomycin resistant *Enterococcus faecium* (VREfm) colonizers, especially characterized by concordant pulsotypes (CP) of paired colonization and infection-related isolates.

**Methods:**

This case-control study was conducted at a teaching hospital between 2011 and 2014. Targeted patients received active surveillance culture for VREfm by anal swabs at admission. Cases were those who developed VREfm infection within 180 days after colonization of VREfm. Controls were those colonized with VREfm without subsequent VREfm infection. CP were defined by similarities ≥86.7% using pulsed-field gel electrophoresis between paired colonization and infection-related isolates.

**Results:**

Ninety-seven cases and 194 controls were enrolled. By conditional multivariable logistic regression analysis, the risk factors for subsequent infection among VREfm colonizers were intensive care unit (ICU) admission (adjusted odds ratio [aOR], 9.32; 95% CI, 3.61–24.02), receipt of central venous catheters (CVC) (aOR, 3.38; 95% CI, 1.30–8.82), and utilization of third- and fourth-generation cephalosporins (aOR, 4.06; 95% CI, 1.79–9.20, and aOR, 5.32; 95% CI, 1.85– 10.29, respectively) (all *P* ≤ 0.01). Fifty-six (57.7%) of case patients belonged to the CP group, which were associated with ICU admission (aOR, 3.74; 95% CI, 1.38–10.13), and infection developing within 30 days after colonization (aOR, 3.34; 95% CI, 1.25–8.91).

**Conclusions:**

Among VREfm colonizers, being admitted to ICU and receiving CVC or broad spectrum cephalosporins, were the risk factors for subsequent infections. These findings highlight the importance of conducting more strict infection control measures on specific groups of VREfm colonizers.

## Background

Enterococci are the top five pathogens causing healthcare-associated infections in the United States [1]. High proportions of clinical enterococcal isolates possess vancomycin resistance, especially *Enterococcus faecium,* ranging from 50.0 to 80.0% [2, 3]. In the US, an estimated 20,000 patients were infected by vancomycin-resistant enterococci (VRE), which were associated with more than 1000 deaths annually in 2013 [4]. In the European Union, population-weighted proportions of VRE infections significantly increased 1.4-fold without geographic difference from 2014 to 2017 [5]. The increasing trends of VRE infections have also been noted in Asia and Oceania [6–9]. Few treatment options and high mortality rates of VRE infections [10], and frequent intra- or inter-healthcare institute spread of VRE [11], all contribute to a huge economic burden for the prevention and management of VRE [12].

Current evidence demonstrates VRE colonization increases risks of subsequent VRE infections [13, 14]. As for VRE colonization, the well-recognized risk factors include exposure to antimicrobials, retention of an invasive device, and contaminated hospital environments [15–17]. Accordingly, specific efforts focusing on infection control and prevention measures, including antimicrobial stewardship, have been proposed to prevent hospitalized patients from acquiring and colonizing VRE.

Not all VRE-colonized patients develop subsequent infections with only 20–45% of these patients developing subsequent VRE infections [13, 14, 18], and risk factors associated with subsequent VRE infections among colonizers are seldom discussed. As for the molecular epidemiology of vancomycin-resistant *E. faecium* (VREfm), prior studies have depicted clonal complex (CC) 17 VREfm as the most prevalent clone, causing colonization and infections among hospitalized patients [19–21]. CC17 VREfm is also the dominant clone disseminating in the environment of healthcare institutes [20]. However, whether the subsequent infection-related VREfm isolates are the same strains as the preceding colonization isolates remains unclear.

Therefore, we conducted the following study to disclose the risk factors associated with the development of subsequent infections among VREfm colonizers, and to clarify whether the subsequent infection was caused by the same VREfm isolate identified in the preceding event of colonization.

## Methods

### Patients

An active surveillance program for VRE was conducted at National Taiwan University Hospital (NTUH), a 2200-bed academic hospital, from January 2011. Anal swabs were obtained from the targeted patients on the day of admission, and sent to the infection control laboratory for surveillance cultures for VREfm. The targeted patients included those admitted to intensive care units (ICU), with underlying hematological malignancy, receiving renal replacement therapy, or residents in long-term care facilities. For those with positive surveillance cultures, infection control bundles for multi-drug resistant pathogens were applied according to Society for Healthcare Epidemiology of America (SHEA) guidelines [22].

From January 2011 to December 2014, patients with the first instance of positive results for active VREfm surveillance were enrolled for follow-up until the development of subsequent infection, death, or 6 months after enrollment. Those with a history of positive culture results of VREfm from clinical specimens prior to active surveillance or developing VREfm infection within 2 days after documentation of colonization were excluded. Among the enrolled patients, those who developed subsequent VREfm infection during the study period were considered as the case patients. The others were considered as the control pool. Selected patients with a 2:1 ratio matched with age and gender to case patients from the control pool were considered as the matched-control patients.

### Data collection and definitions

A standardized case report form was used to collect the demographic, clinical and microbiological data from all case and matched-control patients. All data were collected by reviewing electronic medical records. The status of comorbid conditions was recorded at identification as colonizers. Utilization of given antimicrobial classes were defined as patients taking given antimicrobial agents, either orally or intravenously, for at least three consecutive days. Gastrointestinal intervention was defined as those receiving abdominal surgery, gastrointestinal endoscopy, or drainage from organs or cavities in the abdomen. All healthcare factors and antibiotic utilization were documented within 30 days before the end of follow-up. An episode of VREfm infection was categorized according to the definitions by the Center for Disease Control [23]. Early infection was defined as subsequent VREfm infections developing within 30 days after identification of VREfm colonization.

### Microbiology

The methods for VREfm identification and molecular typing of these isolates were as previously described [7, 24]. In brief, VREfm isolates were identified by bile esculin azide broth containing 8 mg/mL vancomycin (BEAV) and chromogenic agar medium, and the confirmatory identification was performed by using the Vitek2 System (*bioMérieux*, Marcy-l’Étoile, France).

Multilocus sequence typing (MLST) were performed for all available colonization and infection VREfm isolates. Furthermore, the electrokaryotypes of VREfm colonization and infection-related isolates collected from the case patients were analyzed by pulsed-field gel electrophoresis (PFGE) to determine the genetic relatedness of paired colonization and infection isolates from the same patient. PFGE patterns were determined using the Pearson product-moment correlation coefficient, with the Gel Compare II software package (*bioMérieux*, Marcy-l’Étoile, France). An Unweighted-pair group method using average linkages (UPGMA) dendrograms were constructed by these data [5]. The cut-off value of similarity above or equal to 86.7% was used to categorize isolates as the concordant pulsotypes (CP) [25], and the others were grouped as the discordant pulsotypes (DP).

### Statistical analysis

Medians and interquartile ranges were calculated for continuous variables, and percentages for categorical variables. Continuous variables were compared using a Mann–Whitney *U* test, and categorical variables using a χ2 test or a 2-tailed Fisher’s exact test, as appropriate. Conditional logistic regression analyses were performed to analyze the risk factors of subsequent infections among VREfm colonizers. Additionally, the Kaplan-Meier analysis was performed to compare the time to subsequent infections between CP and DP groups among case patients. A log-rank test was used to test the differences between the above two groups at the end of follow-up. Logistic regression analyses were also used to analyze the predictors of the CP group among the case patients. Variables with a *P* value of 0.1 or less in the univariable analysis, or those with potential biological meanings, were included in the multivariable analysis. Multivariable models were developed using a stepwise method, using minimization of the Akaike information criterion (AIC). Following the stepwise AIC selection, only variables with *P* values of 0.05 or less were considered significant and included in the final model. The analyses were performed using Stata software (version 14; StataCorp, College Station, TX). Two-sided *P* values less than 0.05 were considered significant.

## Results

### Clinical characteristics of VREfm colonizers and those with subsequent infections

A total of 2631 patients had positive results for active VREfm surveillance during the study period. Ninety-seven (3.2%) VREfm colonized patients who had subsequent VREfm infections were defined as case patients. One hundred and ninety-four patients matched with age and gender were selected from the control pool as matched control patients.

Table 1 shows the comparisons of demographics and clinical characteristics between the case- and matched-control patients. Compared to matched-control patients, case patients were likely to have a higher Charlson score, moderate-to-severe renal disease, ICU admission, and neutropenia, and newly-received hemodialysis, GI intervention, parental hyperalimentation, and central venous catheters (CVC) (all *P* < 0.05). As for utilization of antibiotics, case patients tended to receive third- or fourth-generation cephalosporins, carbapenems, glycopeptide, fluoroquinolone, and linezolid (all *P* < 0.05). As for subsequent infection, 40 case patients had bloodstream infections, 53 urinary tract infections, 3 surgical site infections, and one intra-abdominal infections.

**Table 1:** Demographics, clinical characteristics, and conditional logistic regression analysis among patients with vancomycin-resistant *Enterococcus faecium* colonization with and without subsequent infections

Variable^a^	Case patients (*n* = 97)	Control patients (*n* = 194)	Univariable OR (95% CI)	*P*	Multivariable OR (95% CI)	*P*
Demographics						
Age (y)	70.1 (57.6–81.3)	72.1 (57.6–82.5)	1.00 (0.98–1.01)	0.57		
Male sex	48 (49.5)	96 (49.5)	1.00 (0.27–3.72)	> 0.99		
Comorbid conditions at identification						
Charlson score	4.0 (3.0–6.0)	3.0 (2.0–5.0)	1.13 (1.01–1.25)	0.03		
Myocardial infarction	4 (4.1)	10 (5.2)	0.79 (0.24–2.61)	0.70		
Congestive heart failure	10 (10.3)	28 (14.4)	0.67 (0.31–1.47)	0.32		
Peripheral occlusive arterial disease	1 (1.0)	8 (4.1)	0.25 (0.03–2.00)	0.19		
Cerebrovascular diseases	17 (17.4)	24 (12.2)	1.50 (0.70–3.21)	0.30		
Hemiplegia	6 (6.2)	16 (8.3)	0.70 (0.25–2.00)	0.51		
Dementia	6 (6.2)	15 (7.7)	0.79 (0.30–2.08)	0.64		
Chronic pulmonary disease	7 (7.2)	13 (6.7)	1.10 (0.38–3.19)	0.60		
Connective tissue disease	3 (3.1)	3 (1.6)	2.38 (0.38–14.97)	0.36		
Peptic ulcer disease	19 (19.6)	29 (14.9)	1.37 (0.73–2.55)	0.33		
Mild liver diseases	14 (4.4)	16 (8.3)	1.83 (0.86–3.88)	0.11		
Moderate-to-severe liver diseases	7 (7.2)	8 (4.1)	1.83 (0.63–5.29)	0.26		
Moderate-to-severe renal diseases	39 (40.2)	53 (27.3)	2.07 (1.15–3.73)	0.02	2.64 (1.00–6.93)	0.05
Diabetes mellitus without end organ damage	35 (36.1)	61 (31.4)	1.27 (0.73–2.19)	0.40		
Diabetes mellitus with end organ damage	7 (7.2)	8 (4.1)	1.83 (0.63–5.29)	0.26		
Solid tumor without metastases	21 (21.7)	47 (24.2)	0.83 (0.42–1.61)	0.58		
Metastatic solid tumor	7 (7.2)	11 (5.7)	1.31 (0.48–3.62)	0.60		
Leukemia	17 (17.5)	28 (14.4)	1.56 (0.62–3.94)	0.35		
Lymphoma	4 (4.1)	13 (6.7)	0.62 (0.20–1.89)	0.40		
Acquired immunodeficiency syndrome	1 (1.0)	0 (0)	NA	NA		
Healthcare factors within 30 days before EOF						
Receipt of solid organ transplantation	0 (0)	4 (2.1)	NA	NA		
Receipt of hematopoietic stem cell transplantation	3 (3.1)	15 (7.7)	0.33 (0.09–1.24)	0.10	0.37 (0.08–1.63)	0.19
New receipt of hemodialysis	15 (15.5)	7 (3.6)	6.46 (2.12–19.69)	0.001	3.66 (0.99–13.73)	0.05
ICU admission	66 (67.0)	43 (22.2)	10.63 (5.03–22.48)	< 0.001	9.32 (3.61–24.02)	< 0.001
GI intervention	13 (13.4)	9 (4.6)	3.34 (1.32–8.47)	0.01		
Steroid use	50 (51.6)	107 (55.7)	0.80 (0.48–1.33)	0.39		
Chemotherapy	18 (18.6)	46 (23.8)	0.65 (0.31–1.34)	0.24		
Parental hyperalimentation	40 (41.2)	54 (28.0)	1.98 (1.14–3.46)	0.02		
Neutropenia	14 (14.4)	10 (5.2)	3.44 (1.37–8.65)	0.009		
Mechanical ventilator	49 (50.5)	90 (46.4)	1.24 (0.71–2.17)	0.45		
Indwelling urinary catheter	38 (39.2)	92 (47.4)	0.68 (0.40–1.16)	0.16		
Central venous catheters	87 (89.7)	120 (61.9)	7.05 (3.11–15.97)	< 0.001	3.38 (1.30–8.82)	0.01
Antibiotics use within 30 days before EOF						
Antipseudomonal penicillin	20 (20.6)	24 (12.4)	1.89 (0.97–3.68)	0.06		
Penicillins combined with β-lactamases inhibitors	2 (2.1)	7 (3.6)	0.54 (0.10–2.79)	0.46		
First-generation cephalosporin	0 (0)	3 (1.6)	NA	NA		
Second-generation cephalosporin	6 (6.2)	5 (2.6)	2.68 (0.74–9.68)	0.13		
Third-generation cephalosporin	32 (33.0)	27 (13.9)	2.95 (1.63–5.32)	< 0.001	4.06 (1.79–9.20)	0.001
Fourth-generation cephalosporin	29 (29.9)	18 (9.3)	4.48 (2.21–9.07)	< 0.001	5.32 (1.85–10.29)	0.002
Carbapenem	40 (41.2)	32 (16.5)	4.35 (2.28–8.30)	< 0.001		
Fluoroquinolone	27 (27.8)	29 (15.0)	2.17 (1.20–3.95)	0.01		
Metronidazole	7 (7.2)	11 (5.7)	1.29 (0.49–3.44)	0.61		
Aminoglycoside	8 (8.3)	13 (6.7)	1.24 (0.50–3.07)	0.64		
Glycopeptide	29 (29.9)	31 (16.0)	2.28 (1.25–4.14)	0.007		
Tigecycline	3 (3.1)	10 (5.2)	0.60 (0.17–2.18)	0.44		
Daptomycin	8 (8.3)	17 (8.8)	0.93 (0.38–2.28)	0.88		
Linezolid	14 (14.4)	8 (4.1)	4.79 (1.70–13.48)	< 0.001		
SXT	9 (9.3)	14 (7.2)	1.36 (0.53–3.46)	0.52		
Others^b^	19 (19.6)	22 (11.3)	2.00 (0.99–4.03)	0.05	0.36 (0.12–1.05)	0.06

*Abbreviations*: *IQR* interquartile range, *OR* odds ratios, *CI* confidence interval, *NA* not applicable, *ICU* intensive care unit, *EOF* end of follow-up, *SXT* Trimethoprim/sulfamethoxazole

^a^Data are median values (interquartile range) for continuous variables and number of cases (percentage) for categorical variables. Mann–Whitney *U* test was used to compare continuous variables, and χ^2^ or Fisher exact test was used to compare categorical variables

^b^Other antimicrobials: numbers of patients receiving of colistin, penicillin, macrolides, and clindamycin were 10, 5, 3, and 3 in case group, and 18, 1, 3, and 0 in control group. A patient may receive more than one antibiotic within 30 days before end of follow-up

By conditional multivariable logistic regression, independent risk factors for developing subsequent infections among VREfm colonizers were intensive care unit (ICU) admission (adjusted odds ratio [aOR], 9.32; 95% CI, 3.61–24.02), receipt of central venous catheters (CVC) (aOR, 3.38; 95% CI, 1.30–8.82), and utilization of third- and fourth-generation cephalosporins (aOR, 4.06; 95% CI, 1.79–9.20, and aOR, 5.32; 95% CI, 1.85–10.29, respectively) (all *P* ≤ 0.01).

### Molecular typing of vancomycin-resistant *Enterococcus faecium* colonization and infection-related isolates

A total of 357 VREfm isolates were collected. There were 97 paired isolates among cases, and only 162 isolates available among the matched controls. Distributions of sequence types of colonization and infection-related isolates in cases, and colonization isolates in controls, were not significantly different (Table 2). Sequence type (ST) 17 was the most prevalent ST among colonization isolates in case and control groups, and among subsequent infection-related isolates, ranging from 34.0 to 42.0%. The rests were genetically related to ST17. Of note, 61 paired isolates from the case group shared the same STs, including 26 (42.6%) belonging to ST17; 13 (21.3%) belonging to ST78; 10 (16.4%) belonging to ST341; 8 (13.1%) belonging to ST414; 1 (1.6%) belonging to ST18; and 4 (6.5%) belonging to three different sequence types (ST203 [*n* = 2], ST671 [*n* = 1], and ST1022 [*n* = 1]). All these STs among paired isolates belonged to the clonal complex 17 (CC17) VREfm.

**Table 2:** Distributions of sequence types and pulsotypes among colonization isolates of cases and controls and subsequent infection isolates of cases

	Case		Control
	Colonization isolates (*n* = 97)	Subsequent infection isolates (*n* = 97)	Colonization isolates (*n* = 162)^a^
Sequence type (ST), N (%)			
ST17	33 (34.0)	39 (40.2)	68 (42.0)
ST18	4 (4.1)	3 (3.1)	11 (6.8)
ST78	16 (16.5)	19 (19.6)	30 (18.5)
ST341	17 (17.5)	15 (15.5)	21 (13.0)
ST414	13 (13.4)	12 (12.4)	11 (6.8)
Others^b^	14 (14.5)	9 (9.3)	21 (13.0)
Pulsotype, N (%)			
1	9 (9.3)	7 (7.2)	NA
16	9 (9.3)	6 (6.2)	NA
6	6 (6.2)	7 (7.2)	NA
28	6 (6.2)	6 (6.2)	NA
23	4 (4.2)	6 (6.2)	NA
Others^c^	63 (64.8)	65 (67.0)	NA

Abbreviation: *NA* not applicable

^a^Only 162 colonization isolates in control group were available

^b^Colonization isolates in case group: ST64, 1 (1.0%); ST80, 1 (1.0%); ST203, 4 (4.1%); ST252, 3 (3.1%); ST1022, 1 (1.0%); ST1023, 1 (1.0%); ST1039, 1 (1.0%); ST1050, 2 (2.1%); Infection isolates in case group: ST64, 2 (2.1%); ST203, 2 (2.1%); ST233, 1 (1.0%); ST262, 2(2.1%); ST927, 1 (1.0%); ST1022, 1 (1.0%); Colonization isolates in control group: ST64, 5 (3.1%); ST80, 1 (0.6%); ST125, 1 (0.6%); ST203, 2 (1.2%); ST233, 3 (1.9%); ST252, 3 (1.9%); ST262, 4 (2.5%); ST267, 1 (0.6%); ST767, 1 (0.6%)

^c^There were 45 and 44 different pulsotypes among colonization and infections isolates in cases, respectively

**Fig. 1:**
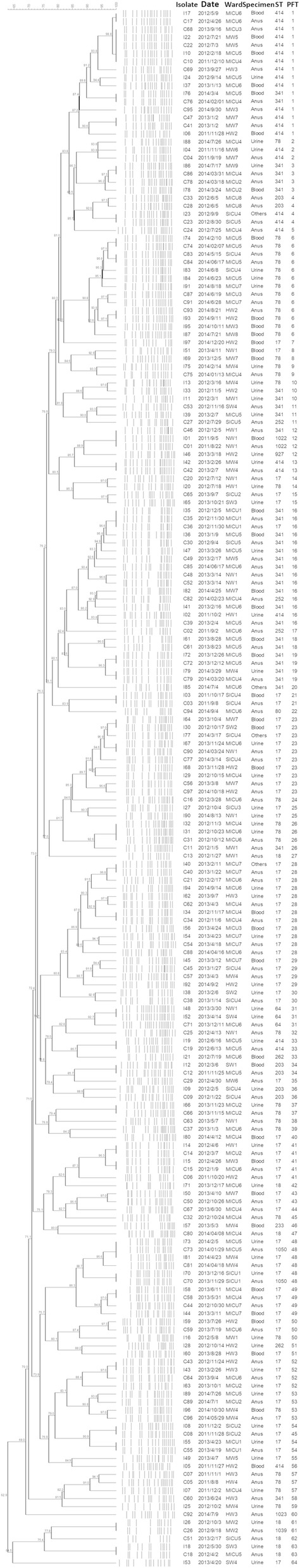
Pulse-field gel electrophoresis of 97 paired vancomycin-resistant *Enterococcus faecium* colonization and infection isolates. Wards of isolate collection were grouped as medical wards (MW), hematology ward (HW), nephrology wards (NW), surgical wards (SW), medical intensive care units (MICU), and surgical intensive care units (SICU), and the number of a certain ward group represents different wards. No., numbers; ST, sequence type; PFT, pulsotype

Ninety-seven paired isolates among cases consisted of 64 pulsotypes (PFT) in 29 groups by PFGE (Fig. 1). Of them, 56 (57.7%) paired isolates showed the concordant pulsotypes (CP) between their colonization and infection-related isolates. These included six pairs (10.7%) belonging to PFT 6, followed by PFT 1 (*n* = 4, 7.1%), PFT 28 (*n* = 4, 7.1%), PFT 2 (*n* = 3, 5.4%), and PFT 48 (*n* = 3, 5.4%). The PFTs of the remaining 36 pairs (64.3%) were very diverse (29 different PFTs).

### Clinical characteristics and predictors for patients with the concordant pulsotypes of vancomycin-resistant *Enterococcus faecium* infections

**Fig. 2:**
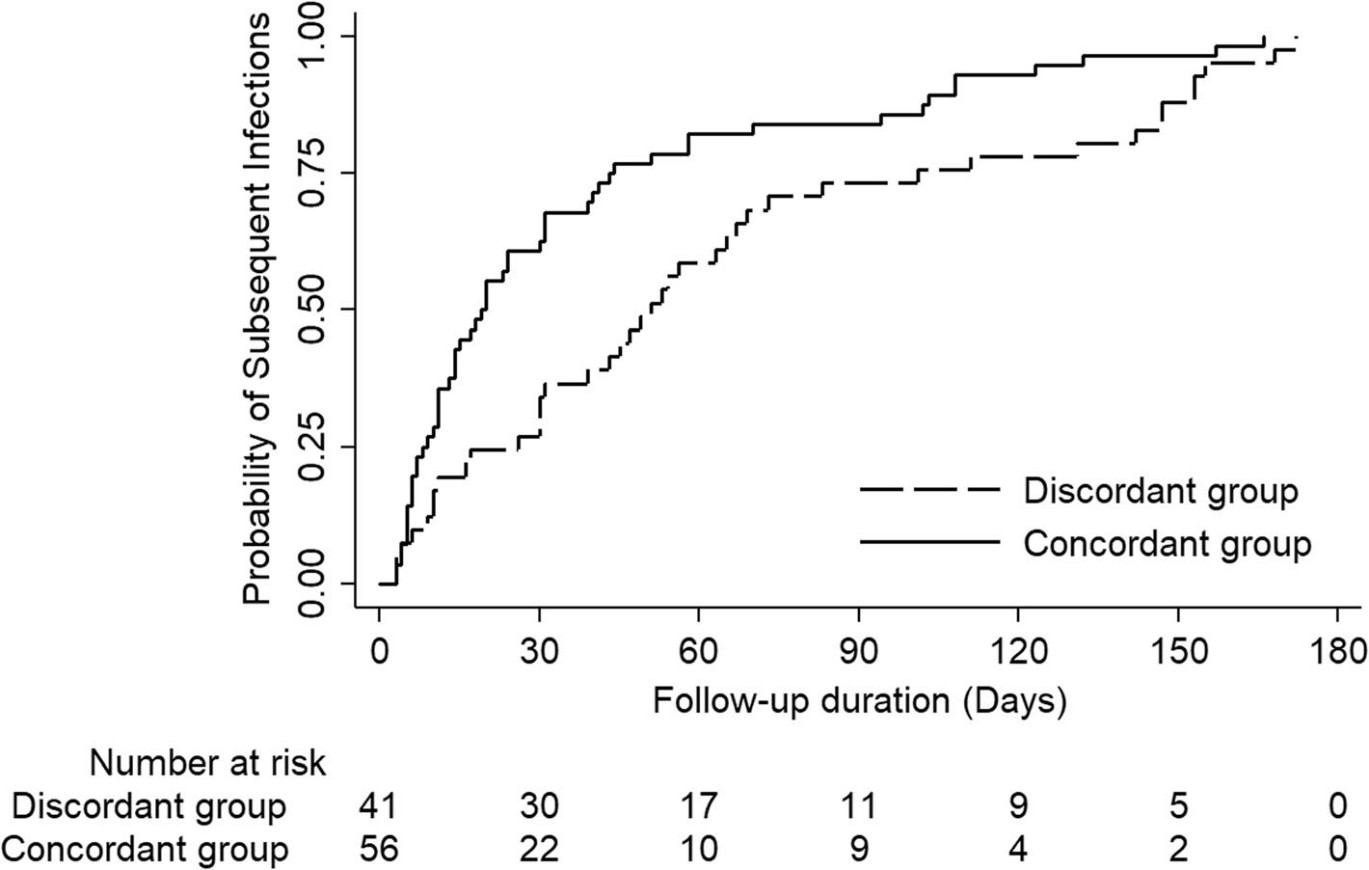
Kaplan–Meier estimates of the time to subsequent infections among vancomycin resistant *Enterococcus faecium* colonized patients, log-rank test *P* = 0.003

Figure 2 shows the cumulative proportions of subsequent VREfm infections in CP and DP groups using a Kaplan-Meier curve. The time to subsequent infections among the CP group was significantly shorter than that among the DP group (long-rank test *P* = 0.003).

As shown in Table 3, by multivariable logistic analysis, predictors for the CP group were ICU admission (aOR, 3.74; 95% CI, 1.38–10.13, *P* = 0.009), early infection (aOR, 3.34; 95% CI, 1.25–8.91, *P* = 0.02), and cerebrovascular disease (aOR, 0.22; 95% CI, 0.06–0.78, *P* = 0.02). No utilization of specific antibiotics was independently associated with the CP group, except fluoroquinolones showing a decreased odds ratio with borderline significance. In addition, common PFTs of colonization isolates, including PFT 1, PFT 6, PFT 16, PFT 23, and PFT 28, were not associated with CP.

**Table 3:** Univariable and multivariable logistic regression analyses of predictors for the concordant pulsotypes of paired vancomycin-resistant *Enterococcus faecium* colonization and infection isolates

Predictors^a^	Patients infected with the concordant pulsotypes (*n* = 56)	Patients infected with the discordant pulsotypes (*n* = 41)	Univariable OR (95% CI)	*P*	Multivariable^c^ OR (95% CI)	*P*
Demographics						
Age (y)	70.9 (60.0–78.6)	65.6 (55.2–77.4)	1.01 (0.98–1.04)	0.56		
Gender	30 (53.6)	18 (43.9)	1.47 (0.66–3.31)	0.35		
Comorbid conditions at identification						
Charlson score	4.0 (2.5–5.0)	4.0 (3.0–6.0)	0.95 (0.81–1.12)	0.55		
Myocardial infarction	3 (5.4)	1 (2.4)	2.26 (0.23–22.59)	0.46		
Congestive heart failure	4 (7.1)	6 (14.6)	0.45 (0.12–1.71)	0.23		
Peripheral occlusive arterial disease	1 (1.8)	0 (0)	NA	NA		
Cerebrovascular diseases	6 (10.7)	10 (24.4)	0.37 (0.12–1.13)	0.08	0.22 (0.06–0.78)	0.02
Hemiplegia	3 (5.4)	3 (7.3)	0.72 (0.14–3.75)	0.69		
Dementia	6 (10.7)	0 (0)	NA	NA		
Chronic pulmonary disease	4 (7.1)	3 (7.3)	0.97 (0.21–4.61)	0.97		
Connective tissue disease	2 (3.6)	1 (2.4)	1.48 (0.13–16.91)	0.75		
Peptic ulcer disease	11 (19.6)	8 (19.5)	1.01 (0.37–2.78)	0.99		
Mild liver diseases	6 (10.7)	8 (19.5)	0.50 (0.16–1.56)	0.23		
Moderate-to-severe liver diseases	5 (8.9)	2 (4.9)	1.91 (0.35–10.38)	0.44		
Moderate-to-severe renal diseases	22 (39.3)	17 (41.5)	0.91 (0.40–2.08)	0.83		
Diabetes mellitus without end organ damage	21 (37.5)	14 (34.2)	1.16 (0.50–2.69)	0.73		
Diabetes mellitus with end organ damage	3 (5.4)	4 (9.8)	0.52 (0.11–2.48)	0.41		
Solid tumor without metastases	11 (19.6)	10 (24.4)	0.76 (0.29–2.00)	0.58		
Metastatic solid tumor	4 (7.1)	3 (7.3)	0.97 (0.21–4.61)	0.97		
Leukemia	8 (14.3)	9 (22.0)	0.59 (0.21–1.70)	0.33		
Lymphoma	2 (3.6)	2 (4.9)	0.72 (0.10–5.35)	0.75		
Acquired immunodeficiency syndrome	1 (1.8)	0 (0)	NA	NA		
Healthcare factors within 30 days before EOF						
Receipt of solid organ transplantation	0 (0)	0 (0)	NA	NA		
Receipt of hematopoietic stem cell transplantation	1 (1.8)	2 (4.9)	0.35 (0.03–4.05)	0.40		
New receipt of hemodialysis	10 (17.9)	5 (12.2)	1.57 (0.49–4.99)	0.45		
ICU admission	45 (80.4)	20 (48.8)	4.30 (1.75–10.56)	0.001	3.74 (1.38–10.13)	0.009
GI intervention	9 (16.1)	4 (9.8)	1.77 (0.51–6.21)	0.37		
Steroid use	26 (46.4)	24 (58.5)	0.61 (0.27–1.38)	0.24		
Chemotherapy	8 (14.3)	10 (24.4)	0.52 (0.18–1.45)	0.21		
Parental hyperalimentation	25 (44.6)	15 (36.6)	1.40 (0.61–3.19)	0.42		
Neutropenia	9 (16.1)	5 (12.2)	1.38 (0.43–4.47)	0.59		
Mechanical ventilator	34 (60.7)	15 (36.6)	2.68 (1.17–6.15)	0.02		
Indwelling urinary catheter	25 (44.6)	13 (31.7)	1.74 (0.75–4.03)	0.20		
Central venous catheters	51 (91.1)	36 (87.8)	1.42 (0.38–5.26)	0.60		
Antibiotics use within 30 days before EOF						
Antipseudomonal penicillin	12 (21.4)	8 (19.5)	1.13 (0.41–3.06)	0.82		
Penicillins combined with β-lactamases inhibitors	0 (0)	2 (4.9)	NA	NA		
First-generation cephalosporin	0 (0)	0 (0)	NA	NA		
Second-generation cephalosporin	4 (7.1)	2 (4.9)	1.50 (0.26–8.60)	0.65		
Third-generation cephalosporin	16 (28.6)	16 (39.0)	0.63 (0.27–1.47)	0.28		
Fourth-generation cephalosporin	18 (32.4)	11 (26.8)	1.29 (0.53–3.15)	0.57		
Carbapenem	24 (42.9)	16 (39.0)	1.17 (0.52–2.66)	0.71		
Fluoroquinolone	9 (16.1)	18 (43.9)	0.24 (0.10–0.63)	0.003	0.35 (0.12–1.02)	0.05
Metronidazole	4 (7.1)	3 (7.3)	0.97 (0.21–4.61)	0.97		
Aminoglycoside	6 (10.7)	2 (4.9)	2.34 (0.45–12.24)	0.31		
Glycopeptide	12 (21.4)	17 (41.5)	0.39 (0.16–0.94)	0.04		
Tigecycline	2 (3.6)	1 (2.4)	1.48 (0.13–16.91)	0.75		
Daptomycin	5 (8.9)	3 (7.3)	1.24 (0.28–5.52)	0.78		
Linezolid	8 (14.3)	6 (14.6)	0.97 (0.31–3.05)	0.96		
SXT	4 (7.1)	5 (12.2)	0.55 (0.14–2.21)	0.40		
Others^b^	9 (16.1)	10 (24.4)	0.59 (0.22–1.63)	0.31		
Infection type						
BSI vs. other types of infection	24 (42.9)	16 (39.0)	1.17 (0.52–2.66)	0.71		
Early infection	36 (64.3)	13 (31.7)	3.88 (1.64–9.12)	0.001	3.34 (1.25–8.91)	0.02
Common PFTs of colonization isolates^d^	18 (32.1)	16 (39.2)	0.74 (0.32–1.72)	0.48		

*Abbreviations*: *IQR* interquartile range, *OR* odds ratios, *CI* confidence interval, *NA* not applicable, *ICU* intensive care unit, *EOF* end of follow-up, *SXT* Trimethoprim/sulfamethoxazole, *BSI* bloodstream infections, *PFTs* pulsotypes

^a^Data are median values (interquartile range) for continuous variables and number of cases (percentage) for categorical variables. Mann–Whitney *U* test was used to compare continuous variables, and χ^2^ or Fisher exact test was used to compare categorical variables

^b^Other antimicrobials: numbers of patients receiving of colistin, penicillin, macrolides, and clindamycin were 3, 4, 2, and 2 in case group, and 7, 1, 1, and 1 in control group. A patient may receive more than one antibiotic within 30 days before end of follow-up

^c^Pearson goodness-of-fit test *P* = 0.5574 > 0.05 (df = 15); Hosmer and Lemeshow goodness-of-fit test *P* = 0.5578 > 0.05 (df = 7)

^d^Common PFTs consist of PFT 1, PFT 6, PFT 16, PFT 23, and PFT 28

## Discussion

As VREfm infection has become one of leading threats in healthcare systems, having a preventive strategy plays an important role, along with limited, effective antibiotics. Thus, identifications of modifiable risk factors for developing VREfm infection is an urgent task, because effective preventive approaches can only be constructed after they are disclosed. Our study not only found clinical predictors for subsequent infections among VREfm colonized patients, but also further elucidated critically ill patients, and those with short spans between colonization and infection status, had a greater risk of being infected by preceding colonized VREfm isolates, confirmed by using molecular typing methods. Of note, our study echoed that most colonization and infection-related VREfm isolates belonged to CC17, a global epidemic clone complex.

Our analysis demonstrated that admission to ICU, and receipt of CVC, and broad-spectrum cephalosporins, including 3rd- and 4th-generation, are the clinical predictors for hospitalized patients from VREfm colonization to infection. These findings are aligned with prior studies identifying several healthcare-associated risk factors, including receipt of broad spectrum antibiotics and presence of hemodialysis catheters [26–28]. All aforementioned findings suggesting antibiotics selection pressure and certain healthcare factors, especially different catheter types, are important triggers for VREfm from colonization to infection. Therefore, our findings support the current evidence that bundle care and antibiotics stewardship are parts of an effective preventive strategy against VREfm infection [22, 29].

Utilization of glycopeptide was a recognized risk factor for acquisition and/or infections of VRE [30, 31]. But our findings and others showed that utilization of glycopeptide didn’t become one of predictors for subsequent VREfm infection in the final multivariable analysis [26, 28]. One possible explanation was that among our targeted patients, more than half of them (51.7%, 31/60) receiving glycopeptide also received 3rd- and 4th-generation cephalosporins during their follow-up periods. The high collinearity precludes these factors from being putting into the same statistical model simultaneously. Therefore, these antibiotics classes might not be considered independently but assumed to affect together [28].

We also investigated whether subsequent VRE infections came from previous anal colonization by whether paired isolates shared a similar or identical genetical relationship defined by PFGE. We found ICU stay was independently associated with concordant paired isolates. In contrast, the findings that patients with CVA were less likely to have concordant paired isolates might be attributed to utilization of less CVC (64.7% vs. 93.8%, *P* < 0.002). Our findings again suggested that more antibiotics selection pressure and indwelling catheters may trigger VREfm from colonization to infection, especially in critically ill patients.

Interestingly, our findings also revealed the shorter span between colonization and infection predicted the risks of concordant VRE paired isolates. Prior studies have shown that the shorter time intervals between paired VRE samplings, either from two colonization events, or from two infection events, the more likely VRE strains are similar/identical [32–34]. Our findings echoed the fact that VREfm colonization in humans is dynamic over time. However, the mechanisms of VREfm dynamics caused by either strain replacement of VREfm colonization, or by introduction of a new strain after resolution of primary VREfm colonization in the same patients, remains unclear. Large follow-up studies in VREfm colonized patients are warranted to demonstrate the potential mechanisms of VREfm dynamics.

As for the molecular typing of VREfm colonized and infection-related isolates, prior investigations have demonstrated CC17 VREfm is the dominant colonized and infection strain. Few studies have depicted CC17 VREfm from colonization to infection by using paired isolates from the same patients [35]. Our study, to our best knowledge, was the largest cohort to demonstrate this relation of CC17 VREfm. However, one major caveat of MLST to trace genetic relatedness of VREfm is that it is less discrimitive, because gene recombination is a common mechanism of genetic variation among VREfm [36]. Our findings, that the numbers of concordant paired isolates defined by PFGE were less than those defined by MLST (numbers of paired isolates: 56 vs. 62), suggested more discriminative power by PFGE compared to MLST in VREfm isolates. This finding further echoes the recommendations that PFGE is a more robust molecular typing method to distinguish the genetic relatedness of different VREfm isolates [37].

There were several limitations in this study. First, selection bias was inevitable in a case-control design. Second, caution should be taken in generalizing our findings given VREfm colonizers in our cohort were only identified through active surveillance by anal swabs among targeted high-risk population in a single center. We did not analyze data of those with VREfm colonization identified by clinical specimens. Third, even though our study lacked a whole genome sequence (WGS) to discriminate the genetic relatedness between colonization and infection isolates, previous studies have demonstrated a high threshold of PFGE similarities, as set in this study, and so have comparably discriminative power to WGS [38].

## Conclusions

Our results suggest ICU stay, receipt of CVC or broad-spectrum antibiotics were the potential driving forces for VREfm from gastrointestinal colonization to infection. More specifically, ICU stay was associated with higher odds for subsequent infections among VREfm colonizers when concordant paired isolates were considered. Additionally, early infection within 30 days after identification of VRE colonization was another indicator for concordant paired isolates. Further investigations are warranted to determine whether specific driving forces exist in this specific patient group. Once such predictors are identified, these findings may be applied to form add-on infection control measurements to prevent VREfm infections among these vulnerable patients.

## Data Availability

The datasets used and/or analysed during the current study are available from the corresponding author on reasonable request.

## Abbreviations

AIC: The Akaike information criterion
aOR: Adjusted odds ratio
CC: Clonal complex
CI: Confidence interval
CP: Concordant pulsotypes
CVC: Central venous catheter
DP: Discordant pulsotypes
ICU: Intensive care unit
MLST: Multilocus sequence typing
PFGE: Pulsed-field gel electrophoresis
PFT: Pulsotypes
SHEA: Society for Healthcare Epidemiology of America
ST: Sequence type
UPGMA: Unweighted-pair group method using average linkages
VRE: Vancomycin-resistant enterococci
VREfm: Vancomycin resistant *Enterococcus faecium*
WGS: Whole genome sequence
